# Long-term assessment of whale shark population demography and connectivity using photo-identification in the Western Atlantic Ocean

**DOI:** 10.1371/journal.pone.0180495

**Published:** 2017-08-17

**Authors:** Jennifer A. McKinney, Eric R. Hoffmayer, Jason Holmberg, Rachel T. Graham, William B. Driggers, Rafael de la Parra-Venegas, Beatriz E. Galván-Pastoriza, Steve Fox, Simon J. Pierce, Alistair D. M. Dove

**Affiliations:** 1 Louisiana Department of Wildlife and Fisheries, New Orleans, LA, United States of America; 2 National Marine Fisheries Service, Southeast Fisheries Science Center, Mississippi Laboratories, Pascagoula, MS, United States of America; 3 Wild Me, Portland, OR, United States of America; 4 MarAlliance, San Pedro, Ambergris Caye, Belize; 5 Ch'ooj Ajauil AC, Cancún, Quintana Roo, México; 6 Utila Whale Shark Research, Utila, Bay Islands, Honduras; 7 Marine Megafauna Foundation, Truckee, CA, United States of America; 8 Georgia Aquarium, Atlanta, GA, United States of America; Shark Research Institute, UNITED STATES

## Abstract

The predictable occurrence of whale sharks, *Rhincodon typus*, has been well documented in several areas. However, information relating to their migratory patterns, residency times and connectivity across broad spatial scales is limited. In the present study photo-identification data is used to describe whale shark population structure and connectivity among known aggregation sites within the Western Central Atlantic Ocean (WCA). From 1999 to 2015, 1,361 individuals were identified from four distinct areas: the Yucatan Peninsula, Mexico (n = 1,115); Honduras (n = 146); northern Gulf of Mexico, United States (n = 112), and Belize (n = 49). Seasonal patterns in whale shark occurrence were evident with encounters occurring in the western Caribbean Sea earlier in the year than in the GOM. There was also a significant sex bias with 2.6 times more males present than females. Seventy sharks were observed in more than one area and the highest degree of connectivity occurred among three aggregation sites along the Mesoamerican Reef. Despite this, the majority of resightings occurred in the area where the respective sharks were first identified. This was true for the WCA as a whole, with the exception of Belize. Site fidelity was highest in Mexico. Maximum likelihood modelling resulted in a population estimate of 2,167 (95% c.i. 1585.21–2909.86) sharks throughout the entire region. This study is the first attempt to provide a broad, regional population estimate using photo-identification data from multiple whale shark aggregations. Our aim is to provide population metrics, along with the description of region-scale connectivity, that will help guide conservation action in the WCA. At a global level, rapidly growing photographic databases are allowing for researchers to look beyond the description of single aggregation sites and into the ocean-scale ecology of this pelagic species.

## Introduction

The whale shark (*Rhincodon typus*) is the world’s largest extant fish and has a cosmopolitan distribution in tropical and warm temperate marine ecosystems [1]. Since its description by Smith in 1828 [2], the whale shark has remained an enigmatic species. Many aspects of its life history, such as age at maturity, longevity, and reproductive cycle are still poorly understood [1, 3–6]. This lack of knowledge has hindered conservation efforts. Based on > 50% reduction in the global population over the last 75 years, the IUCN Red List of Threatened Species recently changed the conservation status of the whale shark from “Vulnerable” to “Endangered” [7]. Additionally, this species benefits from several broad protection plans, including being listed under Appendix II of the Convention on International Trade in Endangered species (CITES). Despite these efforts, a more targeted conservation action requires a better understanding of the distribution, population structure and movement patterns [8].

While the whale sharks’ range is well established, information on the global population structure and movement patterns remains limited. Analyses of mitochondrial [9] and nuclear [10] DNA sequence data revealed shared haplotypes for whale sharks in the Atlantic, Indian and Pacific oceans and demonstrated historical gene flow among these ocean basins. These findings are supported by satellite tracking data which have documented large-scale movements across broad, oceanic expanses [11–13]. Conversely, genetic data also reveal significant differences in haplotype frequencies between the Indo-Pacific and Atlantic regions [9,10] suggesting whale sharks within the Atlantic Ocean, particularly the Gulf of Mexico (GOM) [14], are relatively isolated from conspecifics in the Indian and Pacific oceans.

Elucidating large-scale movement patterns of whale sharks has been a challenging, yet growing field of research. The advent of satellite tracking technology has shed light on whale shark short-term movements but examining long-term patterns (>1 year) has been hampered by a number of factors, such as poor retention and non-reporting of tags [1,15]. Movements of aquatic animals have been monitored through the use of external tags since at least 1653 [16]. Typically, conventional tags are imprinted with a unique identifier/code and contact information (e.g. phone number) to allow the observer to relay resighting data to the investigator. While this method has been successful for tracking movements of many aquatic organisms, in whale sharks this approach has been shown ineffective for population studies, due to tag loss, lack of reporting, and inaccurate reporting [17,18]. However, the use of natural spot patterns has been proven useful for identifying individuals, having utility for movement and population studies.

Natural marks (e.g. fin shape, scars, spot patterns) have been widely used to identify individuals from a wide-range of taxa [19], including several shark species [20–24]. Photo-identification of whale sharks based on skin spot-patterns provides a non-invasive method in which individuals can be monitored throughout their lifetime [25] and thus has utility for movement studies, as well as population modelling [18, 26–29]. The applicability of photo-identification in studying the movements of marine organisms was first demonstrated by Würsig and Würsig [30] using individual-specific markings and/or fin shapes to identify and monitor cetaceans. More recently, photo-based analytical techniques, originally developed for investigating social structure in marine mammals [31], have been successfully applied to whale shark sighting data and used to calculate various population parameters [28,29,32,33].

Whale sharks form predictable aggregations at specific sites throughout their range. In the Western Central Atlantic Ocean (WCA), these aggregations occur off the coasts of Belize, Honduras, Mexico and the United States [18, 32, 34–38]. The ability to reliably encounter whale sharks in close proximity to shore has resulted in the development of ecotourism industries in Isla Holbox, Isla Mujeres, Cancun (Mexico), Gladden Spit (Belize) and Utila (Honduras) [34]. While it has been suggested that ecotourism can adversely impact shark populations [4, 18, 26, 39, 40], it also creates an opportunity for the collection of whale shark data at an unprecedented level. Rather than relying on a few dedicated researchers, every tourist with a camera represents a potential citizen scientist collecting photographic data. Holmberg et al. [26] utilized photographic data collected from a number of sources, including citizen scientists, to estimate survival rate for whale sharks at Ningaloo Marine Park off Western Australia. These efforts led to the creation of online repositories for whale shark photographs obtained by researchers and citizen scientists around the world [25, 26, 41]. The largest of these repositories is the Wildbook for Whale Sharks photo-identification library (www.whaleshark.org), established in 2003, which has over 33,000 whale shark encounters, including over 4,000 photos taken in the WCA region. The objective of this study is to utilize these data to generate population parameters, and summarize long-term movements of whale sharks in the WCA.

## Materials and methods

### Study area

**Fig 1:**
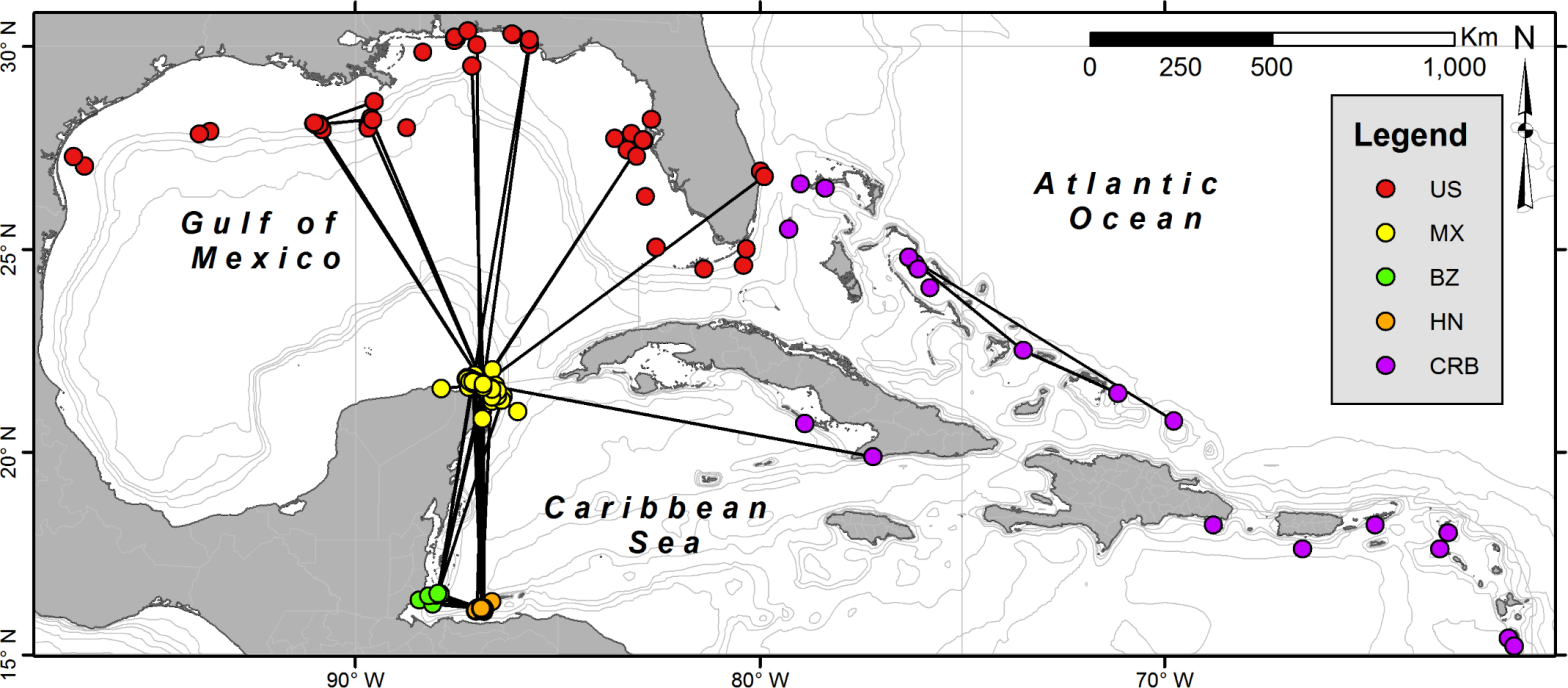
Spatial distribution of sightings data collected through Wildbook for Whale Sharks in the Western Central Atlantic Ocean during 1999–2015. Movements between whale shark sightings within the Gulf of Mexico and Caribbean, including Honduras (HN), Belize (BZ), Mexico (MX), United States of America (US), and the greater Caribbean region (CRB) based on photo-identification data.

The GOM and Caribbean Sea are large, adjacent, and semi-enclosed seas within the WCA (Fig 1). The Caribbean Current, the principal current in the Caribbean Sea, enters the GOM through the Yucatan Channel and forms the Loop Current and the Yucatan Current [42]. These currents serve as major transport mechanisms allowing for regional connectivity for many marine organisms, including invertebrates [43], reef fishes [44–46], and large pelagic teleosts [47]. Within the study region is the Mesoamerican Barrier Reef System, the second largest reef system in the world, which runs from the Honduran Bay Islands along the Belizean coast to the Yucatan Peninsula.

### Standardized photo-identification

Whale shark images used in this study were collected by researchers and recreational divers at various aggregation sites within the WCA, including Utila, Honduras, Gladden Spit, Belize, Yucatan Peninsula, Mexico, in the northern GOM, United States (US), and beyond (Fig 1). Due to the limited number of submissions overall throughout the Bahamian and Caribbean Island chains, this data was omitted from statistical analysis. Photographic identification relies on a clear image of the spot patterning behind the gills (also referred to as the “fingerprint”), on either the left or right sides. When evident in images or specifically noted by person submitting the image, sex was assigned based on the presence (male) or absence (female) of claspers. In addition to images, ancillary data is collected such as sighting date, time, location, and estimated total length. Total length was typically estimated using approximate lengths of nearby boats and divers for scale [18]. Identification of individual sharks was performed using the techniques and computer-assisted matching tools described in Arzoumanian et al. [25]. Individual identifications were assigned to sharks based on having a high-quality spot “fingerprint” coupled by low matching scores with existing sharks and visual assessment by trained researchers. By centralizing the data online, it is possible to make identifications across datasets and geographically distinct research efforts, providing a more cohesive look at spatial and temporal linkages. All whale shark sightings data used in this study are third-party data publicly available on www.whaleshark.org. Data was collected and used according to the Wildbook for Whale Sharks Terms and Conditions, with appropriate permissions. The authors confirm that the data are owned by a third party and that they had no special access privileges to the data sets.

All identification and sighting locations were imported into ArcMap 10.2 (Environmental Systems Research Institute, Inc., Redlands, CA, USA) and projected using the global WGS 1984 PDC Mercator. The minimum straight-line distance (km) between sighting locations was calculated and summed per individual to determine total distance traveled. This was considered to be the minimum distance traveled by each shark, which is an underestimation of true movement. Therefore, spatial error created by paths that may have crossed land was considered insignificant.

### Regional connectivity and population size

The total number of uniquely identified individuals was determined for each area and summed across the entire study region. Since whale sharks are known to aggregate in large numbers, repeated encounters with the same individual within a single calendar month were consolidated to a single occurrence when investigating seasonality of occurrence. A discovery curve was constructed to visualize the number of newly identified individuals per year to determine if abundance estimates can be applied to the dataset [48]. A Student’s t-test was used to determine if there was a significant difference in length between sexes [49]. Differences in sex ratios were also investigated using a chi-square test with Yates’ correction to determine if there was any significant deviation from the assumed 1:1 male to female ratio [49]. These statistical tests were applied to the entire dataset and considered significant at alpha = 0.05. Mean values are reported with standard error throughout.

The residence times for individuals within the study area were investigated using the “movement” module in the compiled version of SOCPROG 2.7 [31]. Individual sighting data were used to estimate Lagged Identification Rate (LIR), the probability that the animal will be sighted again after a variable time lag from its first sighting. This approach, a modification of maximum likelihood methods, has been specifically designed to account for situations where individual identification data have been obtained on an opportunistic basis, and allows for non-random distribution of sampling effort (which, in this case, is focused on known feeding aggregations) [31, 50]. All individuals in the database were included with a location code corresponding to one of the four areas referenced above. Whole-site and within-between site LIRs were estimated and fitted to a series of open and closed population movement scenarios. The model with the lowest quasi-Akaike information criterion (QAIC) value, accounting for over-dispersion of the data, was considered to be the best description of shark residency patterns. The most parsimonious model with 100 bootstraps was used to generate parameter estimates and confidence intervals. Similarly, a movement model was also used to test the probabilities of travel between the four sites within the region, with an additional “outside” option to incorporate sharks that moved away from the primary study areas. In this case, the LIR represented the probability of the individual being sighted again in either the same area or moving to a different area after a specified time lag. In this case, the two most parsimonious model scenarios, fully-mixed and migration with full interchange, were fitted to the data with 100 bootstraps to calculate movement parameters with 95% confidence intervals [50].

## Results

### Western Central Atlantic Whale Shark demographics

**Fig 2:**
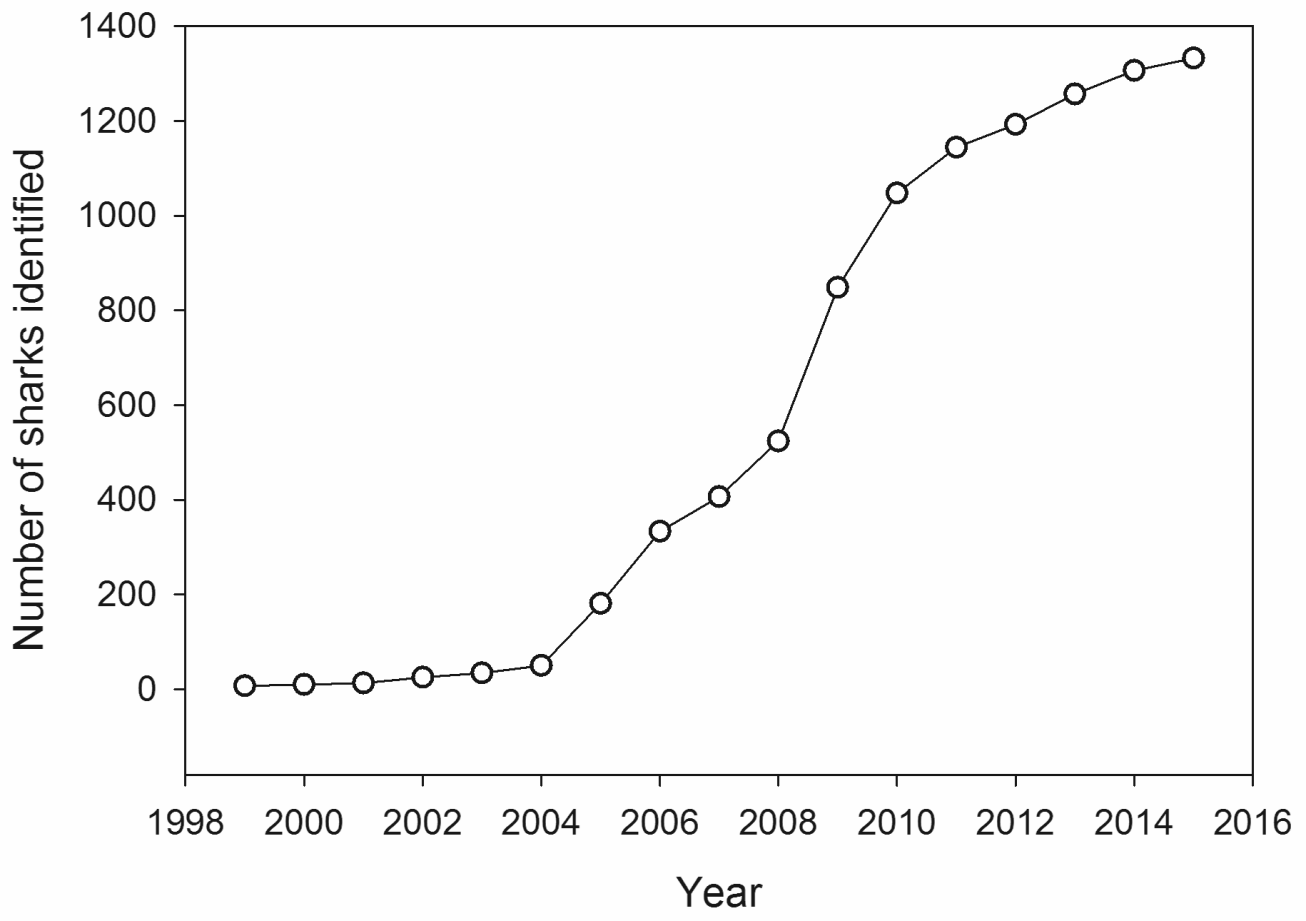
Discovery curve for newly identified whale sharks from the Western Central Atlantic Ocean (1999–2015).

From 1999 to 2015, 4,298 encounters from 1,361 individually identified whale sharks were available for analysis from the WCA (Fig 1). Over 300 people contributed photographic data for analysis, with approximately 82.1% (n = 3,572) of photographs being provided by researchers and 17.9% (n = 777) provided by recreational divers or ‘citizen scientists’. The largest number of identified sharks were obtained from Mexico from 2001–2015 (1,115 sharks during 3,669 encounters), followed by Honduras from 1999–2015 (146 sharks during 337 encounters), United States from 2003–2015 (112 sharks during 123 encounters), Belize from 1999–2015 (49 sharks during 144 encounters), and the greater Caribbean Sea from 2003–2015 (20 sharks during 25 encounters). The number of newly-identified whale sharks per year exhibited no asymptote throughout the study; however, the percentage of resighted individuals remained constant from 2011–2015 at 75.0% (Table 1, Fig 2). Half (n = 683) of the 1,361 sharks identified in this region were reported only once in the database. Of the 678 resighted sharks, 90% (n = 608) were only observed in the area where initially encountered. Only 10% (n = 70) of resighted individuals were observed in more than one area.

**Table 1:** Whale shark photo-identification records for Western Central Atlantic Ocean from 1999–2015 with details of new records and resightings from previous years.

	1999	2000	2001	2002	2003	2004	2005	2006	2007	2008	2009	2010	2011	2012	2013	2014	2015
N	7	6	5	17	13	21	147	190	102	188	483	474	376	235	311	184	90
New	7	3	4	15	10	16	131	152	74	116	326	224	100	46	70	50	26
Resight	0	3	1	5	3	5	16	38	28	72	157	250	276	189	241	134	64
% resight	0.0	50.0	20.0	29.4	23.1	23.8	10.9	20.0	27.5	38.3	32.5	52.7	73.4	80.4	77.5	72.8	71.1

**Fig 3:**
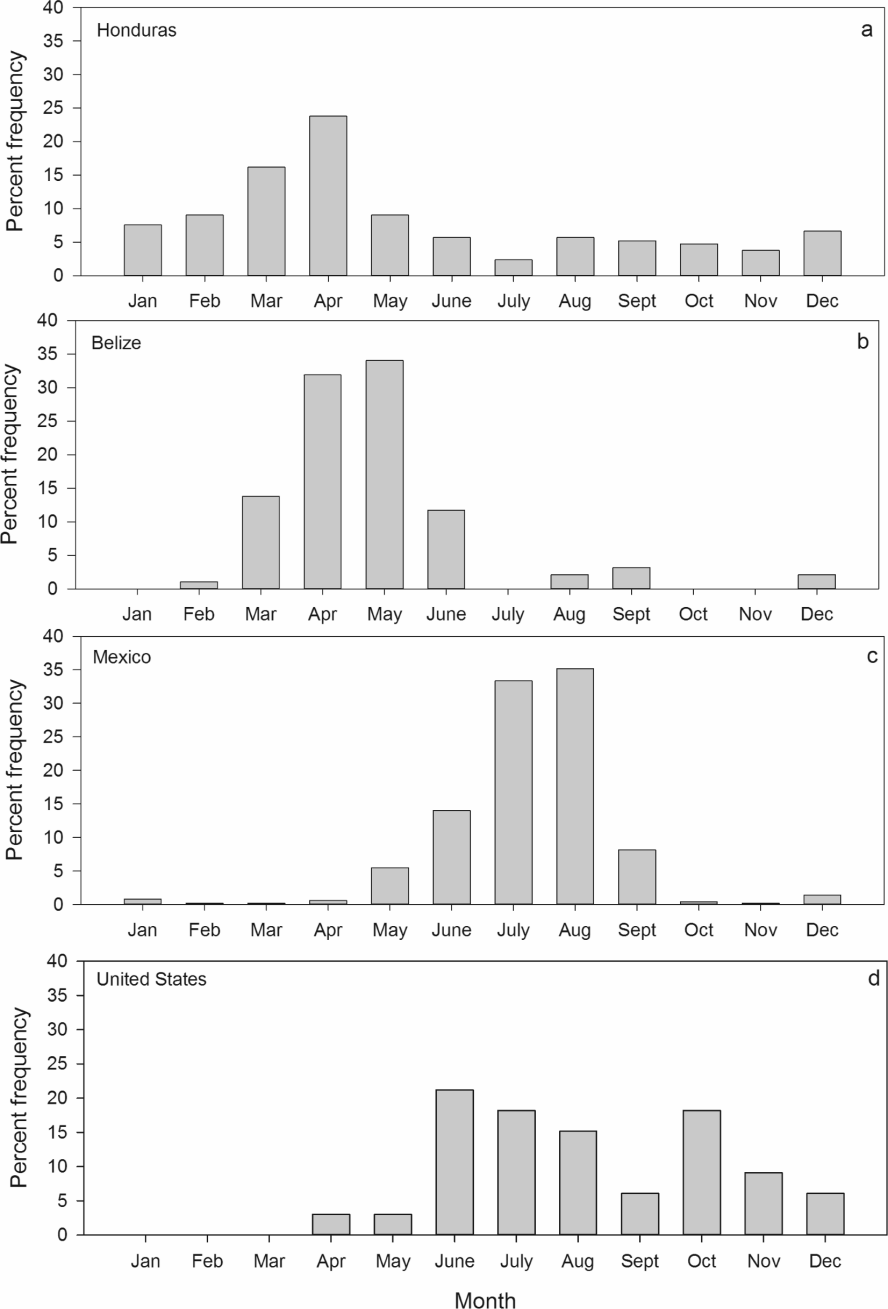
Monthly distribution of whale shark encounters in a) Honduras, b) Belize, c) Mexico, and d) US waters.

A seasonal pattern of whale shark occurrence was evident within the WCA, with encounters occurring off Honduras and Belize earlier in the year than off Mexico and the United States (Fig 3). The highest percentage of encounters occurred during spring off Honduras (57.1%, March to May) and Belize (86.6%, April to June), whereas off Mexico the peak time was primarily during summer (91.7%, June to Sept) (Fig 3A–3C). In US waters, the peak time for whale shark encounters was more protracted, occurring during summer and fall (78.0%, June to October) (Fig 3D).

**Fig 4:**
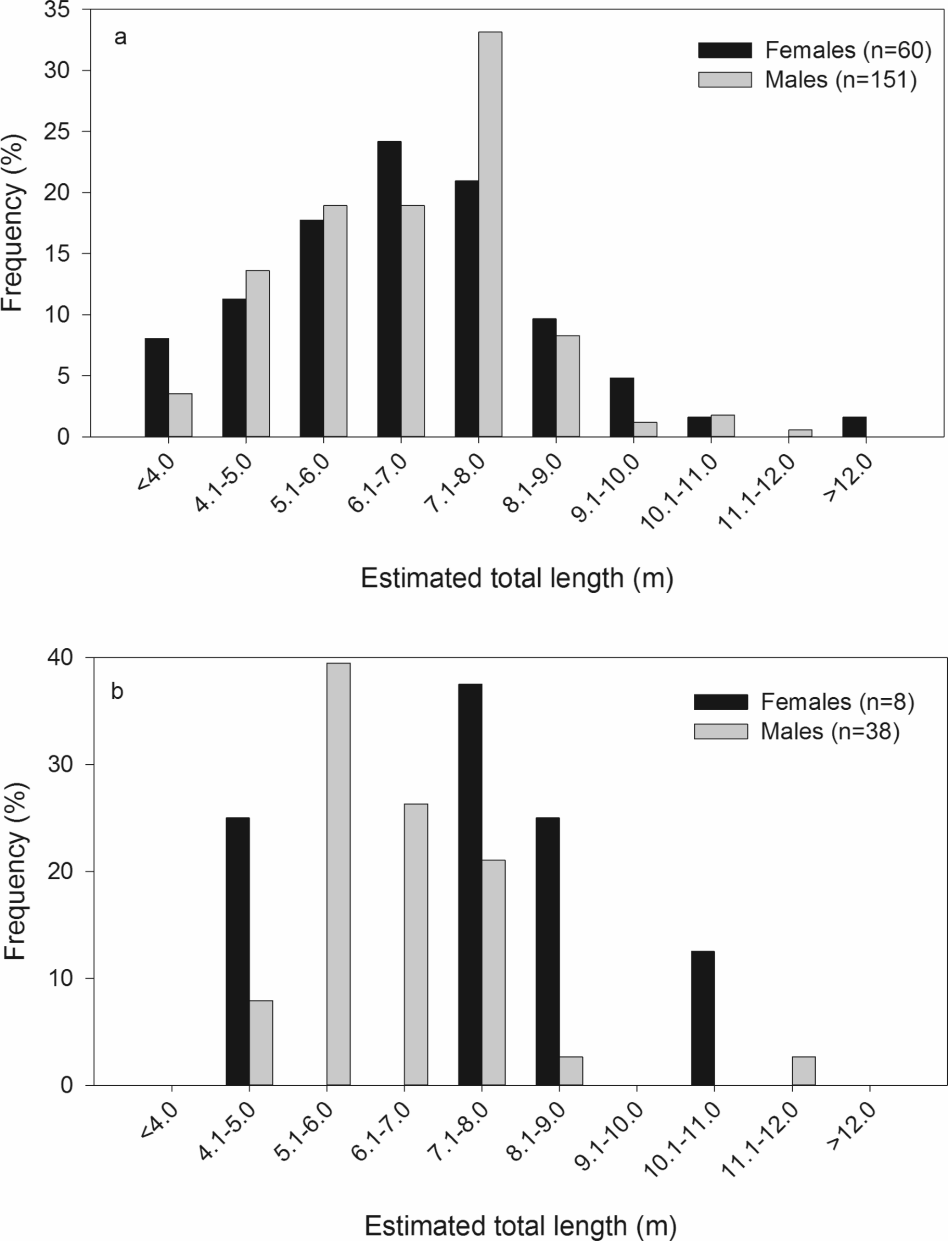
Length frequency distribution for a) all identified individuals and b) individuals that exhibited movement (n = 70) within the Western Central Atlantic region from 1999–2015.

There was a significant sex bias with 2.6 times more males present than females (χ^2^ = 135.2, n = 707, p < 0.001). Estimated total length for whale sharks ranged from 1.8 to 12.1 m, with males ranging from 1.8 to 11.0 m (6.8 ± 0.1 m, n = 169) and females ranging from 2.4 to 10.6 m (6.7 ± 0.2 m, n = 61). There was no significant difference in estimated total length between sexes (t_228_ = 0.148, p = 0.883). Approximately 89% of the whale sharks were estimated to be smaller than 8.0 m (Fig 4A).

### Regional connectivity and population size

**Fig 5:**
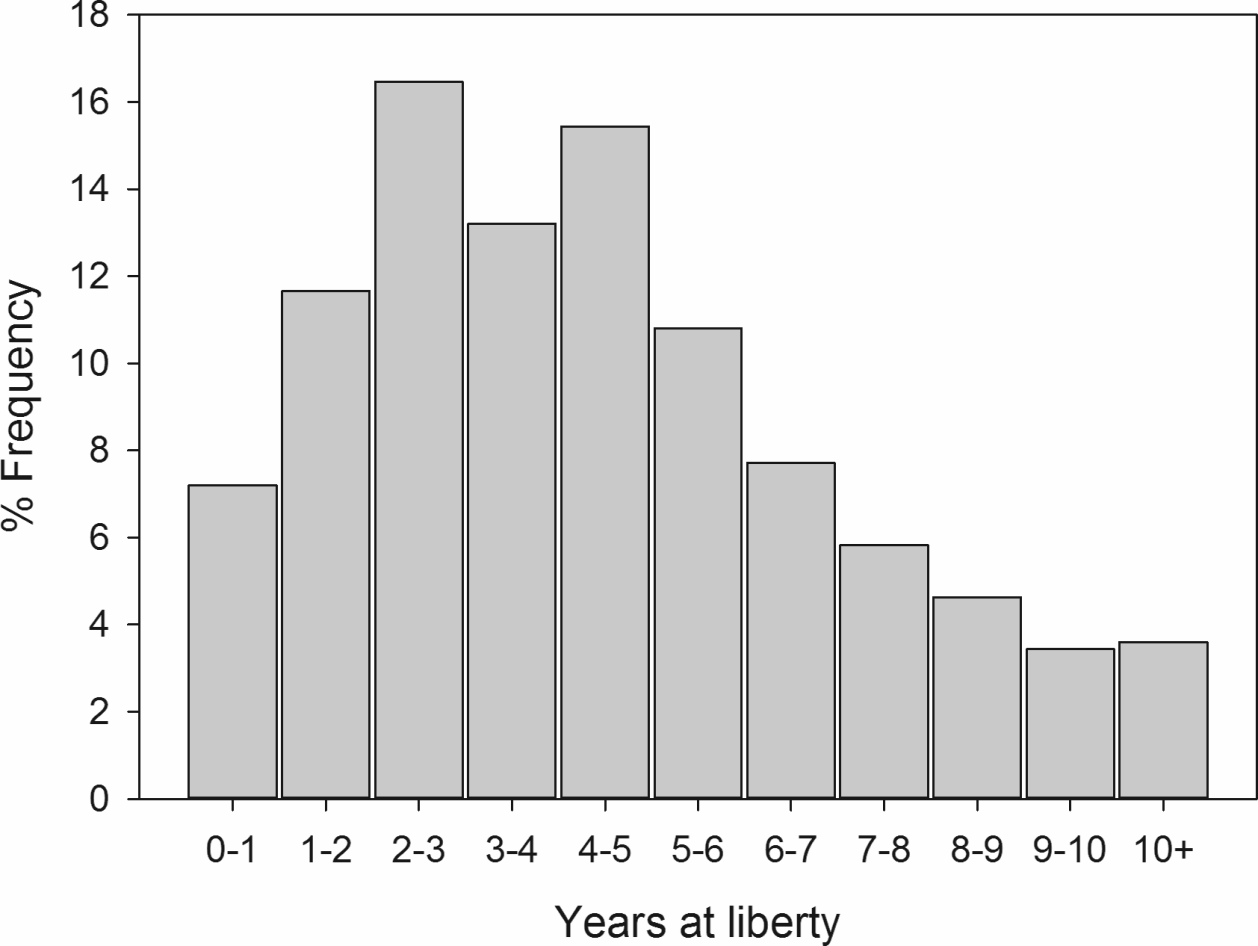
Time-at-liberty (years) distribution of whale sharks in the Wildbook for Whale Sharks photo-identification library from the Western Central Atlantic Ocean.

The highest degree of connectivity occurred among the three aggregation sites along the Mesoamerican Reef, with the majority of the movements documented between Honduras and Belize (n = 40), Honduras and Mexico (n = 39), and Belize and Mexico (n = 18) (Fig 1). The time at liberty for the resighted sharks ranged from 0.1 to 16.5 years and peaked between 2–4 years (1.8 ± 2.6 years) (Fig 5). Sixteen sharks had sighting records longer than 10 years (Table 2). The longest time between sightings of the same individual was 16.5 years (BZ-008), which was first observed in Belize in 1999, resighted in Belize again in 2002, then observed in Mexico in 2008, 2011, 2013, 2014, and 2015. In some cases, sharks were observed repeatedly in the same area for several days to months, and in other cases, many years passed between encounters (Table 2). There was also a time lag evident for sharks observed during the same year among regions along the Mesoamerican Reef. The mean lag time between sharks observed in Belize and Honduras was 26 ± 4.7 days (3–35 days), whereas the lag time between Belize/Honduras and Mexico was 4.4 ± 0.3 months (2–6 months). In almost every case, when sharks were observed in both Belize/Honduras and Mexico in the same year, they were observed in Belize or Honduras during spring and in Mexico during summer. There were 13 movements observed between the United States and Mexican waters, with no apparent seasonal pattern between the movements. In the same year, some sharks moved from Mexico to the United States (MX-030; 2008), whereas others made the reverse transition (MX-343; 2010).

**Table 2:** Summary statistics of whale sharks tracked for over 10 years via photo-identification records in the Western Central Atlantic Ocean from 1999–2015. Lag refers to the time period between sightings. Country abbreviations include: Honduras (HN), Belize (BZ), Mexico (MX), and United States of America (US).

Marked ID	Number of Encounters	First Year	Last Year	Duration (years)	Minimum Lag (days)	Maximum Lag (years)	Mean Lag (years)	Countries Visited
BZ-001	15	2002	2015	12.76	4	4.12	0.91	HN, BZ, MX
BZ-002	7	2002	2013	11.13	13	6.38	1.85	BZ, MX
BZ-007	6	2003	2014	11.32	285	6.37	2.26	BZ, MX
BZ-008	7	1999	2015	16.47	388	6.40	2.75	BZ, MX
BZ-010	7	2003	2014	11.24	31	4.05	1.72	BZ, US, MX
BZ-011	7	1999	2014	15.05	29	5.02	2.51	BZ, HN
BZ-012	3	2003	2014	11.30	1109	8.27	5.65	MX, BZ
BZ-014	5	2003	2013	11.03	1	7.88	2.76	BZ, HN
BZ-021	7	2000	2013	13.18	306	6.13	2.20	BZ, HN
BZ-026	5	2000	2014	13.44	70	9.11	3.36	BZ, MX, HN*
H-006	13	2001	2014	13.00	1	5.73	1.08	BZ, HN
H-017	9	2002	2015	13.25	1	10.25	1.66	BZ, HN, MX
H-021	12	2000	2013	14.22	1	4.87	1.29	BZ, HN, MX, US
H-035	12	1999	2014	15.66	1	7.49	1.42	HN, BZ, MX
MXA-043	7	2002	2015	12.97	37	5.03	2.16	MX
MXA-115	10	2002	2013	10.95	6	7.02	1.22	MX

*indicates observed in Roatan, Honduras rather than Utila, Honduras.

Minimum, maximum, and mean lag calculated as the time between resightings.

**Fig 6:**
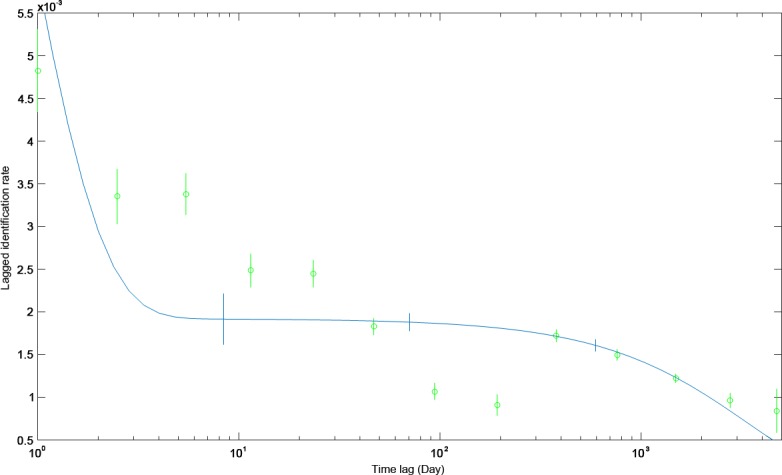
Probability of resighting an individual whale shark over time (LIR; mean +- S.D.) within the Western Central Atlantic compared to the best fitting model (dark line).

Modelled LIR declined rapidly during the first 200 days following initial sighting (Fig 6) There was a slight increase in probability of reidentification at one year (Fig 6), suggesting an annual periodicity of whale shark presence in the study area. The most open model accounting for emigration, reimmigration, and mortality (Model G) best fit the empirical data based on the QAIC (Table 3). In this modeled scenario, there were 57.4 ± 62.6 S. E. (95% c.i. 28.4–277.2) sharks likely to be observed on any given day. The mean residency was 0.8 ± 7.2 S.E. (95% c.i. 0.5–29.7) days within the study area followed by 6.9 ± 8.8 S.E. (95% c.i. 5.1–36.1) days outside of the surveyed area.

**Table 3:** Model comparisons for lagged identification rate of whale sharks throughout the entire study area (A-H) and within/between areas (I-L).

**Model**	**Description for Whole Study Area**	**QAIC**
A	Closed	242037.60
B	a_1_ = N	98990.68
C	Emigration/mortality	98603.14
D	Closed: emigration + reimmigration	98651.35
E	a1 = N; a2 = Mean residence	98603.14
F	a1 = N; a2 = Res time in; a3 = Res time out	98651.35
G	a1 = N; a2 = Res time in; a3 = Res time out; a4 = Mort	98549.83*
H	Emigration + reimmigration + mortality	102325.06
	**Description for Within/Between Areas**	
I	Fully mixed (1/a1 = N)	5321.79*
J	Fully mixed (a_1_ = *N*)	11292.77
K	Migration—full interchange (a_1_ = diffusion rate from area 1 to area 2; a2 = 1/N)	11294.77
L	Migration—full interchange (a_1_ = N; a_2_ = mean residence time in area 1)	5323.64

*indicates model selected for bootstrapping

**Fig 7:**
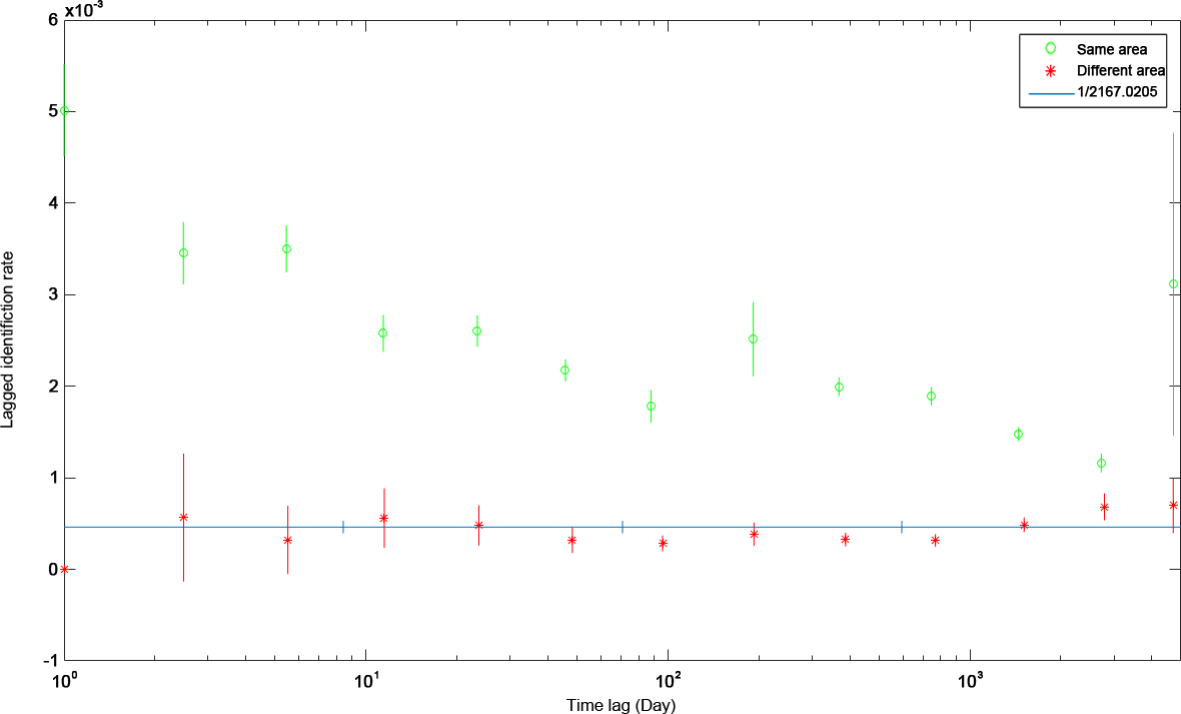
Probability of resighting an individual whale shark over time (LIR; mean ± S.D.) within the same location (green circle) or a different location (red asterisk) in the Western Central Atlantic compared to the best fitting model (dark line).

The LIR was also used to investigate movement within and between the four focal areas in this study, with the addition of a hypothetical “outside” location to account for movement into unmonitored areas. The fully mixed model (Model I; Table 3) best fit the empirical data and generated a population estimate of 2,167 (± 378) (95% c.i. 1695.91–3207.76) sharks. The LIR for resightings in the area in which the shark was originally identified, was much greater than the probability of the shark being resighted within a different area. Resighting probabilities showed the same rapid decline over the first year after sighting with a slight increase at close to a year (Fig 7). After two years, annual LIR showed a slight increase for a probability for resighting in a different area and a steady decrease for the same area. Animals originally observed in Mexico had a 90.8% probability of resighting at the same location; whereas in the other areas, the probability of resighting was approximately 50% (Table 4). In fact, the probability of an individual moving outside of the areas modeled was greater (55.3%) than the resighting probabilities for Honduras (43.4%), Belize (54.5%), and the US (49.2%). Movement between Honduras and Belize was 11% and 12% from these locations to the US. Besides being seen again in the same area, moving to an “outside” area had the highest transition probability for the US (26.8%), Honduras (16.4%), and Mexico (6%). Whale sharks coming from the “outside” have the highest probability of transition to Mexico (19.4%) or the US (11.2%).

**Table 4:** Transition probabilities amongst aggregations sites in the Western Central Atlantic.

			To Area			
		Honduras	Belize	Mexico	USA	Outside
	Honduras	**43.4%**	11.1%	16.8%	12.3%	16.4%
	Belize	11.9%	**54.5%**	15.6%	12.0%	6.1%
**From Area**	Mexico	0.1%	0.0%	**90.8%**	3.1%	6.0%
	USA	3.3%	6.6%	14.1%	**49.2%**	26.8%
	Outside	1.8%	0.0%	19.4%	11.2%	**67.6%**

## Discussion

Whale sharks within the Atlantic Ocean appear to be functionally separate from other populations worldwide [14]. We provide the first WCA regional population estimate for this species of 2,167 (± 345) sharks using maximum likelihood models. Based on our findings, a significant proportion of the WCA whale shark population has been sampled with photo-identification. Although the discovery curve did not reach an asymptote, the resighting rate remained over 70% for the last five years of the study, which is typically indicative of high site fidelity and a relatively small population size [51]. Yet for whale sharks, we are reporting one of the largest population sizes in the literature. In the Indian Ocean, resightings rates were considerably lower than reported here (10–32%) and inversely correlated with population size [52]. The number of individuals identified in this study represented 63% of the modelled population estimate and thus reasonably support this conclusion. The results of this study demonstrate that whale sharks within the WCA represent an open population with significant emigration and re-immigration (incorporating permanent emigration), which is not surprising considering their large size and movement capabilities of this species [28, 32, 33, 40]. Previous studies have shown open population models are the most appropriate for population estimates of whale sharks from photo-identification data [41, 53] and the lack of asymptote in the discovery curve, as we observed, indicates that new animals are entering the region [28, 29]. Given the vast available habitat for whale sharks in the WCA, it is entirely plausible that additional aggregation sites occur within the area that have not been identified by researchers or tourism. Mexico and the U.S. had higher percentages of connectivity with the hypothetical “outside” area than Honduras or Belize, indicating that sharks from the broader region are more likely to be identified in the northern Caribbean and GOM than in the southern Mesoamerican Reef area.

Our modelled population estimate is comparable to previous site-specific studies inside [18, 32, 40] and outside [26, 29, 54] the WCA. For example, mark-recapture models at Holbox Island, Mexico predicted an overall population of 521–809 sharks with annual periodicity [40]. At Gladden Spit, Belize, 106 individuals were identified with a mean sightings rate of 4–6 sharks per day but mark-recapture population estimates could not be calculated [18]. In Utila, Honduras, daily estimates of 4.6 sharks were reported, but a total population estimate was not provided [32]. In comparison, the modelled whale shark population estimate (2,837 sharks ± 1,243.9) for the Arabian Gulf was similar to our study, although the proportion of the population sampled was far smaller (15%) during the four years of the study [29]. Smaller population estimates are reported for Ningaloo Reef in Western Australia (320–440 individuals) [54], and for the Seychelles (348–488 individuals) [17].

Population estimates generated from volunteer sightings data should be taken with a bit of caution for several reasons. First, the uneven distribution in effort throughout the range and the high resighting rate, particularly in Mexico, is indicative of sampling biases that can impact the overall estimate. Due to the anecdotal nature of the dataset, it is impossible to account for heterogeneity of capture probability. Whale shark photo-identification data collection is facilitated by the ecotourism industry, and therefore these data are inherently biased by the seasonality of tourism, weather, and distance from shore. Tourism biases are most likely evident in the Yucatan Peninsula, which supports the largest whale shark ecotourism industry in the world [55] and comprised the largest number of encounters in the present study. There is no doubt that the coast of Mexico supports one of the largest aggregations in the world; however, the tourism industry sponsors (almost daily survey) trips by dedicated researchers. Other areas cannot match this level of survey effort. While imagery provided by “citizen scientists” have been shown to be effective for use in mark-recapture modeling for population estimates [53], our study indicates that dedicated initiatives at each aggregation site are needed to spread awareness, encourage participation, and often collect/submit photos to the database.

Year-round monitoring, outside of peak aggregation times and tourism season, is likely confounded by whale sharks inhabiting waters at greater distances from shore and sea conditions that prevent underwater photography. Cagua et al. [56] demonstrated with acoustic telemetry that sightings data could miss year-round residency due to individuals utilizing subsurface waters, or moving further from shore. Honduras is the only country in the WCA to consistently report whale shark sightings for every month of the year; however, anecdotal accounts of year-round whale shark presence are prevalent in all four known aggregation sites in the WCA. Photo-identification data fails to recognize important habitat areas that do not have tourism or directed research effort. For example, satellite telemetry has shown the Bay of Campeche to be a high use area by whale sharks [13], yet no photo-captures were available from that area. Also, Belize is underrepresented in the current study (n = 47 sharks), as earlier work identified 106 individual sharks [18]; however, equipment failure resulted in the loss of digital images prior to the inclusion in the Wildbook system (R. Graham *pers*. *comm*.). It is therefore possible that the results reported herein are an underrepresentation of true population size. Despite these caveats, photo-identification has proven useful for providing the first regional population estimate with the best available data.

Adequate conservation measures rely on an understanding of the full reproductive stock, which is typically unavailable at individual aggregation sites. The vast majority of whale shark data available is derived from juvenile male-dominated aggregation sites, with few exceptions [1]. Even the sexually integrated population in the Red Sea does not represent a reproductive population because the animals are immature [28]. This is also true for the entire WCA, which is dominated by males and 89% of the sampled individuals were smaller than size of maturity estimates (7.0–8.0 m) [5, 40]. A recent study using stereo-video photogrammetry concluded visual size-estimates of whale sharks tends to underestimate larger individuals and overestimate smaller ones [57]. The ecology described here could change as more accurate methods of measuring marine megafauna become prevalent. Regardless, both the smallest and largest whale sharks tend to be absent from most aggregations, including those in the WCA. The scattered reports of neonatal and mature whale sharks in the Atlantic tend to be associated with mid-oceanic islands [13, 58–62] which receive little research or tourism. Until those demographics can be more reliably surveyed, scientific understanding and management of the species will be biased towards aggregating sub-adults.

Connectivity among whale shark aggregations in the WCA has been well-demonstrated using satellite telemetry, conventional tags, and photo-identification [13, 18, 63]. Yet, movement studies of whale sharks are inherently limited by current methods. Deployment times for satellite tags rarely exceeds one year [64–66], and sightings data is limited by visibility bias and constraints on survey effort [56]. In the present study, the repeated long-distance movements of at least 1,600 km (between Honduras/Belize and the US Gulf Coast), were documented in two individuals. Additionally, movements between Mexico and the northern Gulf of Mexico aggregation site, were documented thirteen times. Other studies have reported multinational connectivity, but usually on a smaller spatial scale [29, 67]. It is difficult to compare movements from photo-identification to telemetry studies because, like conventional tags, only the distance between two locations is known. However, this methodology is most useful for long temporal scales, where electronic tracking technology is limited. With the current technology, it is clear that international cooperation is needed for effective research and management of whale shark populations. Information gathering can only improve as new methods and technologies are developed and researchers at individual aggregation sites continue to work collaboratively.

### Conclusion

We provided the first regional population estimate and provided further support for the high degree of connectivity among whale shark aggregations sites in four countries within the WCA over a 16-year period. Broad-scale movements within this region have been corroborated by the available short-term tagging data (conventional and satellite); however, multi-year periodicity of whale shark occurrence can only currently be elucidated using long-term photo-identification. Despite the inherent biases of opportunistic, observational data, photo-identification has proven to be a useful tool to determine demography, movement, site fidelity, and habitat use of whale sharks over large spatial and temporal scales. Whale shark ecotourism has bolstered the photo-identification process allowing both “citizen scientists” and researchers to contribute to a global database. This paper demonstrates the usefulness of multinational participation and collaboration with regard to research and conservation efforts for whale sharks in the WCA.
